# Preoperative HALP and LMR predict disease-free survival in stage III colon cancer and enable nomogram-based risk stratification

**DOI:** 10.3389/fonc.2026.1825101

**Published:** 2026-04-29

**Authors:** Jiayan Xu, Fei Qu, Xiaoyu Wang, Shuang Liang, Dongying Jiang, Zhenghua Wang

**Affiliations:** 1 Department of Oncology, The First Affiliated Hospital of Jinzhou Medical University, Jinzhou, Liaoning, China; 2 Department of Hospital Infection Management, The Obstetrics & Gynecology Hospital of Fudan University, Shanghai, China; 3 Department of Infection, The First Affiliated Hospital of Jinzhou Medical University, Jinzhou, Liaoning, China

**Keywords:** disease-free survival, HAlP, immunonutritional status, lymphocyte-to-monocyte ratio, nomogram, prognosis, stage III colon cancer, systemic inflammatory markers

## Abstract

**Background:**

Colorectal cancer remains a major cause of cancer-related mortality worldwide. Despite curative resection and standard adjuvant chemotherapy, patients with stage III colon cancer remain at considerable risk of recurrence, with marked survival heterogeneity within the same pathological stage. Systemic inflammatory and immunonutritional biomarkers, including the hemoglobin–albumin–lymphocyte–platelet (HALP) score, systemic immune-inflammation index (SII), and lymphocyte-to-monocyte ratio (LMR), may reflect host–tumor interactions. However, their combined diagnostic and prognostic value in stage III colon cancer patients have not been fully established.

**Methods:**

This retrospective study included 210 patients with stage III colon cancer who underwent curative resection and 220 comparable patients with benign colonic lesions. Diagnostic performance was assessed using receiver operating characteristic analysis and logistic regression. Patients with stage III disease were randomly assigned to training and validation cohorts (7:3). Independent predictors of disease-free survival (DFS) were identified using Cox regression, and a nomogram was constructed and internally validated using the concordance index (C-index), time-dependent ROC analysis, calibration curves, and decision curve analysis.

**Results:**

HALP, SII, and LMR showed moderate discrimination for malignancy, with AUC of 0.773, 0.758, and 0.739, respectively. Multivariable analysis identified HALP (HR = 0.384, 95% CI: 0.225–0.655), LMR (HR = 0.483, 95% CI: 0.286–0.815), tumor stage (HR = 2.435, 95% CI: 1.432–4.140), and chemotherapy cycles (HR = 0.380, 95% CI: 0.223–0.647) as independent predictors of DFS. The nomogram demonstrated good discrimination (C-index 0.759 and 0.743 in the training and validation set) with satisfactory calibration and clinical net benefit.

**Conclusion:**

Preoperative HALP and LMR independently predict DFS in stage III colon cancer. A nomogram integrating inflammatory biomarkers with clinicopathological variables enables individualized recurrence risk estimation and may inform postoperative risk-adapted management.

## 1 Introduction

Colon cancer remains one of the most common malignancies worldwide and is a leading cause of cancer-related mortality (1). Stage III colon cancer is characterized by regional lymph node involvement and carries a substantial risk of recurrence despite radical surgery and standardized adjuvant chemotherapy (2, 3). Although pathological staging forms the foundation of prognostic assessment, considerable heterogeneity in clinical outcomes exists among patients within the same stage (4, 5). This variability highlights the need for additional biomarkers that reflect host–tumor interactions beyond conventional anatomical classification (6, 7).

Tumor progression is not solely determined by intrinsic genetic alterations but is profoundly influenced by the systemic inflammatory environment and the nutritional and immune status of the host (8). Chronic inflammation promotes tumorigenesis through cytokine signaling, angiogenesis, platelet activation, and immune suppression (9). Conversely, lymphocyte-mediated immune surveillance plays a crucial role in controlling micrometastatic disease. Therefore, composite inflammatory indices derived from peripheral blood tests have attracted increasing attention as practical and cost-effective prognostic markers (10).

The lymphocyte-to-monocyte ratio (LMR) reflects the balance between antitumor immune activity and tumor-promoting monocyte/macrophage precursors (11). The systemic immune-inflammation index (SII), incorporating neutrophils, platelets, and lymphocytes, represents overall inflammatory burden (12). The hemoglobin–albumin–lymphocyte–platelet (HALP) score integrates anemia status, nutritional reserve, immune competence, and platelet-mediated tumor activity into a single composite parameter (13).

Previous studies frequently combined patients across different TNM stage when evaluating prognostic biomarkers, potentially introducing substantial baseline heterogeneity (14). Patients with stage I–II disease generally have favorable survival outcomes and distinct therapeutic goals, whereas stage IV disease is characterized by metastatic dissemination, limited curative opportunities, and fundamentally different treatment strategies (15). Pooling these biologically and clinically disparate populations may obscure stage-specific prognostic dynamics and lead to biased effect estimation.

To enhance clinical interpretability and methodological rigor, the present study deliberately focused on patients with stage III colon cancer who underwent curative resection followed by standardized adjuvant chemotherapy. This population represents a critical transitional stage in which recurrence risk remains considerable despite radical treatment, and therapeutic decision-making is particularly sensitive to individualized risk assessment (16). By restricting the cohort to this relatively homogeneous yet high-risk group, our study aims to provide more precise prognostic evaluation and generate findings with stronger translational relevance for optimizing postoperative management.

Although these indices have been individually associated with outcomes in colorectal cancer, their combined diagnostic performance and specific prognostic relevance in stage III colon cancer remain insufficiently investigated (17, 18). Furthermore, few studies have incorporated these inflammatory markers into clinically applicable predictive models.

Accordingly, this study aimed to evaluate the diagnostic value of preoperative HALP, SII, and LMR in distinguishing malignant from benign colonic disease, and develop and internally validate a nomogram integrating inflammatory indices and clinicopathological factors to predict disease-free survival in patients with stage III colon cancer. Given that treatment-related variables may influence outcomes, the model was designed to reflect postoperative prognostic assessment in a real-world clinical context.

## 2 Materials and methods

### 2.1 Study design and patient selection

This retrospective single-center study consecutively enrolled patients who underwent curative-intent resection for stage III colon cancer at our institution between May 2018 and December 2021. For diagnostic evaluation, patients with benign colonic lesions (including polyps and adenomas) treated during the same period were included as a control group. Baseline demographic characteristics, including age and gender, were broadly comparable between the two groups, as summarized in **Table 1**, thereby minimizing potential selection bias and enhancing the validity of subsequent comparative analyzes.

**Table 1:** Baseline clinical and laboratory characteristics of patients with stage III colon cancer and benign colonic lesions.

Characteristics	Benign (n=220)	Malignant (n=210)	χ^2^/t/z	*P*
Age			0.167	0.683
≤65	107(48.6%)	98(46.7%)		
>65	113(51.4%)	112(53.3%)		
Gender			0.798	0.372
Male	134(60.9%)	119(56.7%)		
Female	86(39.1%)	91(43.3%)		
Hemoglobin (g/L)	139.78 ± 14.81	121.26 ± 18.40	11.459	*<*0.001
Lymphocyte count (×10^9^/L)	1.71 ± 0.60	1.48 ± 0.54	4.417	*<*0.001
Monocyte count (×10^9^/L)	0.39(0.31-0.50)	0.44(0.35-0.54)	3.483	*<*0.001
Neutrophil count (×10^9^/L)	3.79 ± 1.64	5.01 ± 2.65	5.717	*<*0.001
Platelet count (×10^9^/L)	203.70 ± 49.19	260.37 ± 74.94	9.224	*<*0.001
Albumin (g/L)	41.92 ± 3.57	38.69 ± 4.06	8.737	*<*0.001
HALP	48.67 ± 20.26	30.80 ± 15.25	10.367	*<*0.001
SII	416(310-563)	767(509-1241)	9.261	*<*0.001
LMR	4.20 ± 1.68	2.86 ± 1.38	9.005	*<*0.001

Clinical and laboratory data were extracted from electronic medical records, including demographic characteristics, clinicopathological variables, complete blood count parameters, biochemical indices, and tumor marker levels. Peripheral venous blood samples were obtained in the fasting state on the morning after admission and prior to any therapeutic intervention to minimize treatment-related confounding. Tumor staging was determined according to the 8th edition of the American Joint Committee on Cancer (AJCC) staging system (19).

### 2.2 Inclusion and exclusion criteria

#### 2.2.1 Inclusion criteria

1) Histopathologically confirmed stage III colon adenocarcinoma; 2) Underwent curative-intent surgical resection; 3) Did not receive neoadjuvant chemotherapy or radiotherapy; 4) Received postoperative adjuvant chemotherapy with the XELOX regimen (oxaliplatin 130 mg/m² intravenously on day 1 plus capecitabine 1000 mg/m² orally twice daily on days 1–14 of a 3-week cycle); 5) Had complete clinicopathological and follow-up data available.

#### 2.2.2 Exclusion criteria

1) A history of other malignancies; 2) Received immunotherapy or targeted therapy; 3) Acute or chronic infectious diseases; 4) Severe systemic inflammatory, metabolic, or hematologic disorders that could influence peripheral blood parameters.

Preoperative bowel obstruction was defined as the inability of a colonoscope to traverse the tumor with radiological or endoscopic evidence of proximal bowel dilatation.

### 2.3 Definitions of inflammatory and immunonutritional indices

The indices were calculated as follows: HALP = hemoglobin (g/L) × serum albumin (g/L) × lymphocytes (×10^9^/L)/platelets (×10^9^/L); SII = platelets (×10^9^/L) × neutrophils (×10^9^/L)/lymphocytes (×10^9^/L); LMR = lymphocytes (×10^9^/L)/monocytes (×10^9^/L).

These indices were selected because they integrate parameters reflecting systemic inflammation, immune competence, anemia, and nutritional status.

### 2.4 Follow-up and study endpoints

Patients were followed through outpatient visits and structured telephone interviews. The final follow-up date was December 31, 2024.

DFS was defined as the interval from the date of curative resection to the first documented tumor recurrence or death from any cause, whichever occurred first. OS was defined as the interval from surgery to death from any cause. The median follow-up duration for patients with stage III colon cancer was 41 months (interquartile range, 36–51 months). During the follow-up period, a total of 93 patients experienced disease recurrence or death and were counted as DFS events. In addition, 24 deaths were recorded for overall survival (OS) analysis.

### 2.5 Statistical analysis

All statistical analyzes were performed using SPSS version 26.0 (IBM Corp., Armonk, NY, USA) and R software version 4.3.1 (R Foundation for Statistical Computing, Vienna, Austria).

Continuous variables were expressed as mean ± standard deviation for normally distributed data and as median (interquartile range) for non-normally distributed data. Comparisons between groups were performed using Student’s t-test or one-way analysis of variance for normally distributed variables and the Mann–Whitney U test or Kruskal–Wallis test for non-normally distributed variables. Categorical variables were compared using the χ² test or Fisher’s exact test, as appropriate.

Receiver operating characteristic (ROC) curve analysis was used to assess the diagnostic performance of HALP, SII, and LMR. The area under the curve (AUC) and corresponding 95% confidence intervals (CIs) were calculated. Optimal cutoff values were determined using the Youden index. Binary logistic regression analysis was performed to identify independent diagnostic predictors.

For prognostic analysis, patients with stage III colon cancer were randomly assigned to training and validation cohorts in a 7:3 ratio using computer-generated random numbers. Kaplan–Meier survival curves were constructed and compared using the log-rank test. Univariate Cox proportional hazards regression was performed to screen potential predictors of DFS. Variables with *P* < 0.10 in univariate analysis and clinically relevant covariates were entered into a multivariable Cox regression model using a backward stepwise likelihood ratio method. Hazard ratios (HRs) and 95% confidence intervals (CIs) were reported. The proportional hazards assumption was evaluated using Schoenfeld residuals. No significant violations were observed. To assess potential multicollinearity among variables, variance inflation factors (VIFs) were calculated, and variables with VIF<5 were considered acceptable.

The model incorporated both baseline clinicopathological factors and treatment-related variables to reflect postoperative prognostic assessment. A nomogram for predicting DFS was constructed based on independent predictors identified in multivariable analysis. Model discrimination was evaluated using the concordance index (C-index) and time-dependent ROC curves. Internal validation was conducted using 1000 bootstrap resamples to reduce overfitting bias. Calibration curves were generated to assess agreement between predicted and observed survival probabilities. Decision curve analysis (DCA) was performed to evaluate clinical net benefit across a range of threshold probabilities.

All statistical tests were two-sided, and *P* < 0.05 was considered statistically significant.

### 2.6 Ethical considerations

This study was conducted in accordance with the Declaration of Helsinki and was approved by the Institutional Ethics Committee of The First Affiliated Hospital of Jinzhou Medical University (Approval Number: 2024048). Given the retrospective design and use of anonymized data, the requirement for written informed consent was waived.

## 3 Results

### 3.1 Baseline characteristics and inflammatory profiles

A total of 651 patients were initially screened for eligibility. After applying the predefined inclusion and exclusion criteria, 112 patients were excluded due to missing data, 37 due to neoadjuvant therapy, 21 due to a history of other malignancies, and 51 due to incomplete follow-up. Finally, 210 patients with stage III colon cancer and 220 patients with benign colonic lesions were included in the analysis. A total of 430 participants were included in the present study, comprising 210 patients with pathologically confirmed stage III colon cancer and 220 patients with benign colonic lesions. Baseline demographic, clinical, and laboratory characteristics are summarized in **Table 1**.

There were no statistically significant differences between the two groups with respect to age, sex distribution, or body mass index. However, marked differences were observed in systemic inflammatory and nutritional parameters. Compared with patients with benign lesions, those with malignant disease exhibited significantly lower hemoglobin and albumin levels, reduced lymphocyte counts, and elevated neutrophil and platelet counts. Consequently, HALP and LMR values were significantly decreased, whereas SII values were significantly increased in the cancer cohort (*P* < 0.001).

These findings indicate that stage III colon cancer is accompanied by a distinct systemic inflammatory and immunonutritional profile, suggesting that peripheral blood–derived indices may reflect underlying tumor-related biological alterations.

### 3.2 Diagnostic performance of HALP, SII, and LMR

To evaluate the diagnostic potential of inflammatory indices, ROC curve analysis was performed to distinguish malignant from benign colonic disease.

**Figure 1:**
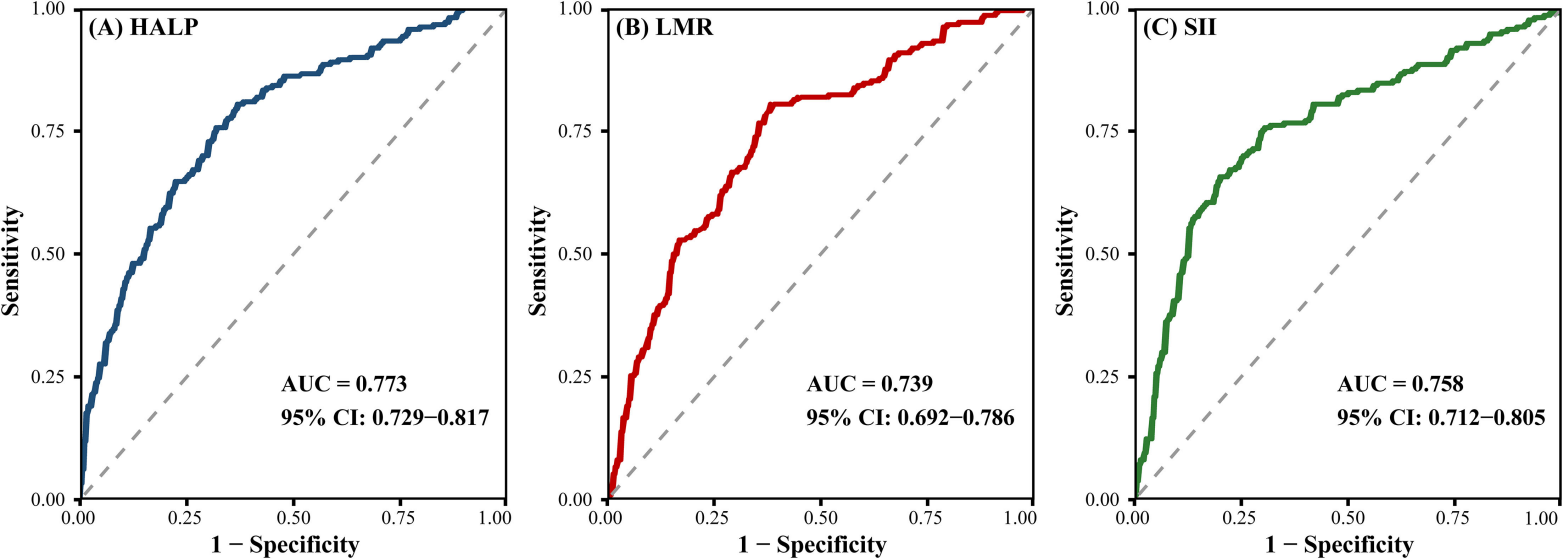
Diagnostic performance of preoperative inflammatory indices for distinguishing stage III colon cancer from benign colonic lesions. **(A)** HALP, **(B)** LMR, and **(C)** SII.

In exploratory analyzes, HALP demonstrated the highest discriminatory ability, with the AUC of 0.773. SII and LMR yielded AUC values of 0.758 and 0.739, respectively. Optimal cutoff values were determined using the Youden index, and corresponding sensitivity and specificity values are detailed in **Table 2**. ROC curves are illustrated in **Figure 1**.

**Table 2:** Diagnostic performance of preoperative inflammatory indices in distinguishing stage III colon cancer from benign colonic lesions.

Diagnostic marker	AUC	95%CI	Cut-off value	Sensitivity	Specificity	Accuracy
HALP	0.773	0.729-0.817	38.31	68.20%	75.70%	71.90%
SII	0.758	0.712-0.805	607.46	65.70%	80.00%	73.00%
LMR	0.739	0.692-0.786	3.77	61.80%	80.50%	70.90%

Cutoff values were determined using the Youden index.

Overall, all three indices exhibited moderate diagnostic accuracy, with HALP showing the most balanced performance. Logistic regression analysis further supported the independent association between inflammatory indices and malignant disease.

### 3.3 Associations with tumor characteristics and survival outcomes

The association between preoperative inflammatory indices and tumor burden, as well as clinical outcomes, was further evaluated in patients with stage III colon cancer.

Low HALP levels were significantly associated with advanced T stage and lymph node involvement, suggesting a link between impaired systemic condition and tumor invasiveness. Elevated SII values correlated with bowel obstruction and higher overall tumor stage, indicating that heightened inflammatory activity accompanies greater tumor burden. Reduced LMR levels were significantly associated with unfavorable pathological characteristics, consistent with compromised immune surveillance and enhanced tumor-promoting inflammatory signaling. Detailed subgroup analyzes are presented in **Supplementary Tables S1**-**S3**.

Kaplan–Meier survival analysis demonstrated that patients with low HALP, high SII, and low LMR experienced significantly shorter DFS. Regarding OS, only low LMR retained a statistically significant association with poorer outcome. Kaplan–Meier curves for individual inflammatory markers are presented in **Supplementary Figure S1**.

### 3.4 Independent prognostic factors for disease-free survival (in training set)

To determine whether inflammatory indices independently predicted postoperative recurrence, univariate Cox regression analysis was performed. The proportional hazards assumption was evaluated using Schoenfeld residuals, and no significant violations or time-dependent effects were detected (seen **Supplementary Figure S2**).

HALP, LMR, tumor stage, and chemotherapy cycles were significantly associated with DFS in univariate analysis. These variables were subsequently entered into multivariable Cox regression.

After adjustment for potential confounders, low HALP, low LMR, advanced tumor stage, and fewer chemotherapy cycles remained independent predictors of poorer DFS, whereas SII did not retain statistical significance, suggesting potential overlap with other inflammatory parameters. For clarity, **Table 3** presents the key variables retained in the final multivariable model, while the full univariate and multivariable Cox regression results for all evaluated covariates are provided in **Supplementary Table S4**.

**Table 3:** Univariate and multivariable Cox regression analyzes for disease-free survival in patients with stage III colon cancer (training set).

Covariates	Univariate analysis			Multivariate analysis		
	HR	95% CI	*P*	HR	95% CI	*P*
Tumor Stage (IIIC vs IIIA-IIIB)	2.474	1.460-4.191	<0.001	2.435	1.432-4.140	0.001
Number of Chemotherapy Cycles (≥6 vs <6)	0.347	0.204-0.590	<0.001	0.380	0.223-0.647	<0.001
HALP (>24.11 vs ≤24.11)	0.286	0.172-0.476	<0.001	0.384	0.225-0.655	<0.001
LMR (>2.32 vs ≤2.32)	0.371	0.224-0.614	<0.001	0.483	0.286-0.815	0.006

These findings indicate that selected inflammatory indices offer prognostic information beyond conventional staging.

### 3.5 Development and validation of the prognostic model

**Figure 2:**
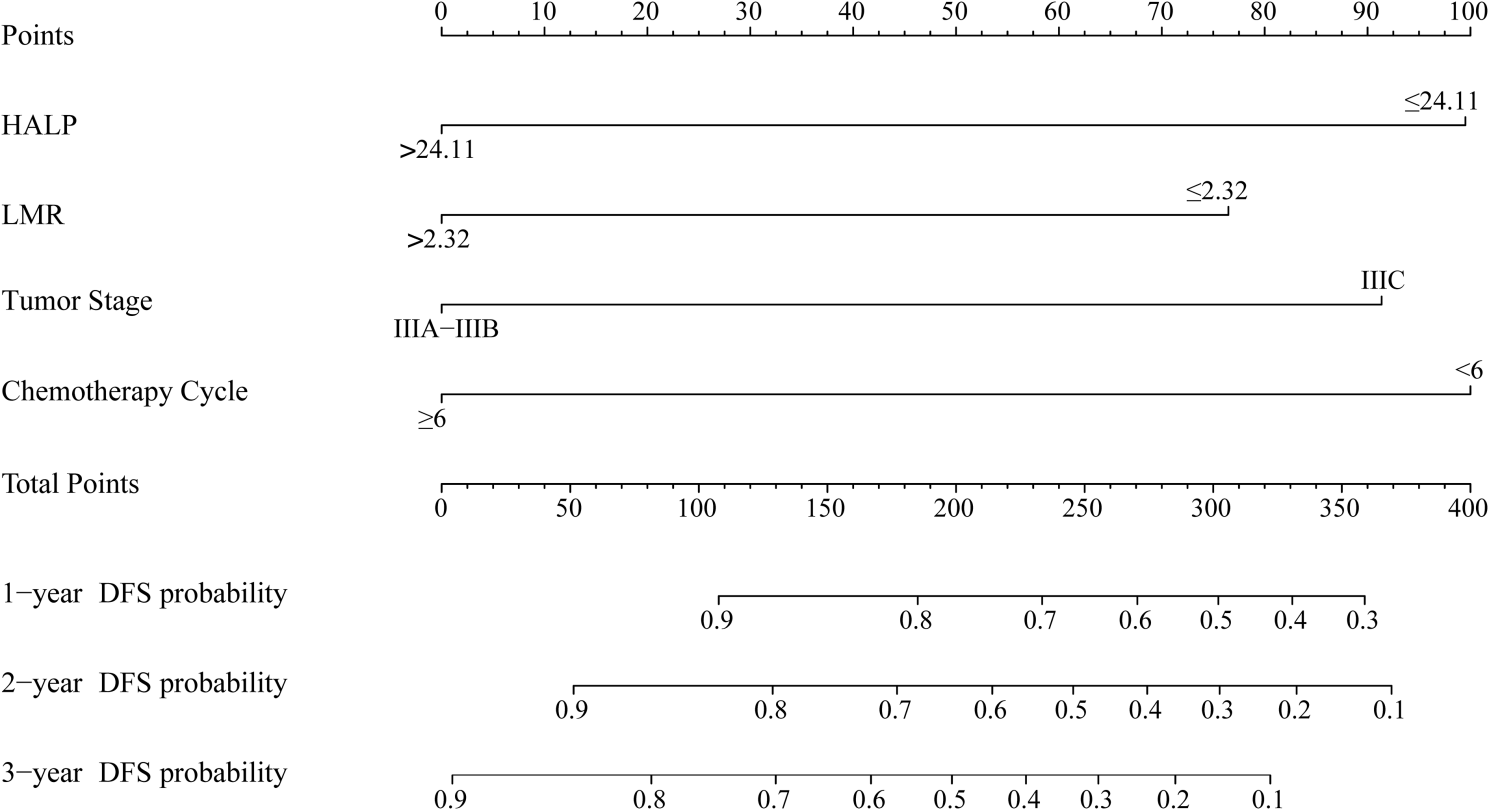
Nomogram for predicting 1-, 2-, and 3-year disease-free survival in patients with stage III colon cancer.

Based on independent predictors identified in multivariable analysis, a nomogram incorporating HALP, LMR, tumor stage, and chemotherapy cycles was constructed to estimate individualized 1-, 2-, and 3-year DFS probabilities. The nomogram is shown in **Figure 2**.

In the training cohort, the model achieved a concordance index (C-index) of 0.759, indicating good discrimination. Internal validation in the independent validation cohort yielded a C-index of 0.743, demonstrating stable predictive performance.

**Figure 3:**
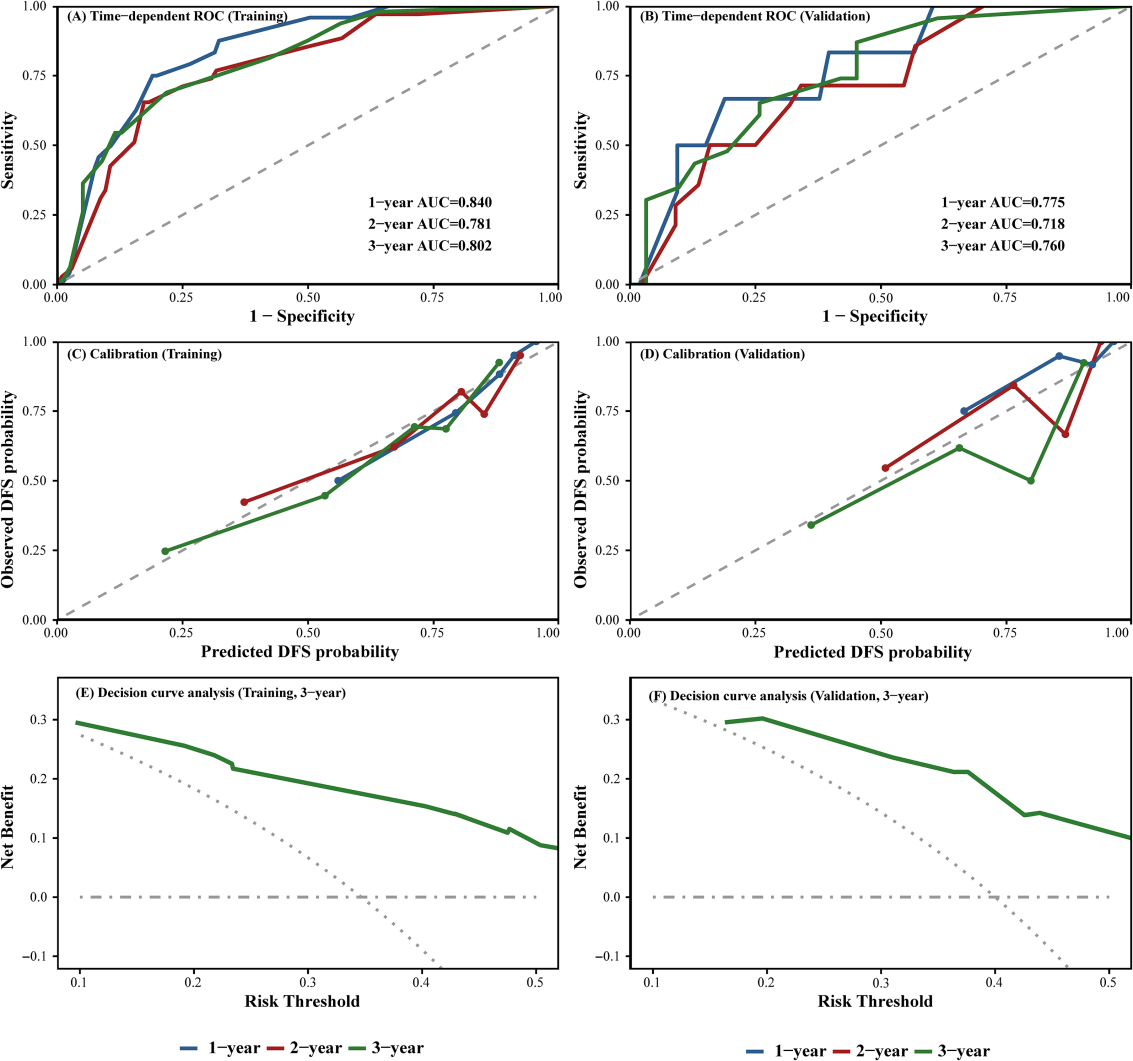
Performance of the prognostic model in the training and validation cohorts.

Time-dependent ROC analysis confirmed consistent accuracy at 1, 2, and 3 years. Calibration plots revealed good agreement between predicted and observed survival probabilities. DCA demonstrated meaningful net clinical benefit across a range of threshold probabilities, supporting the practical utility of the model. Validation results are illustrated in **Figure 3**.

**Figure 4:**
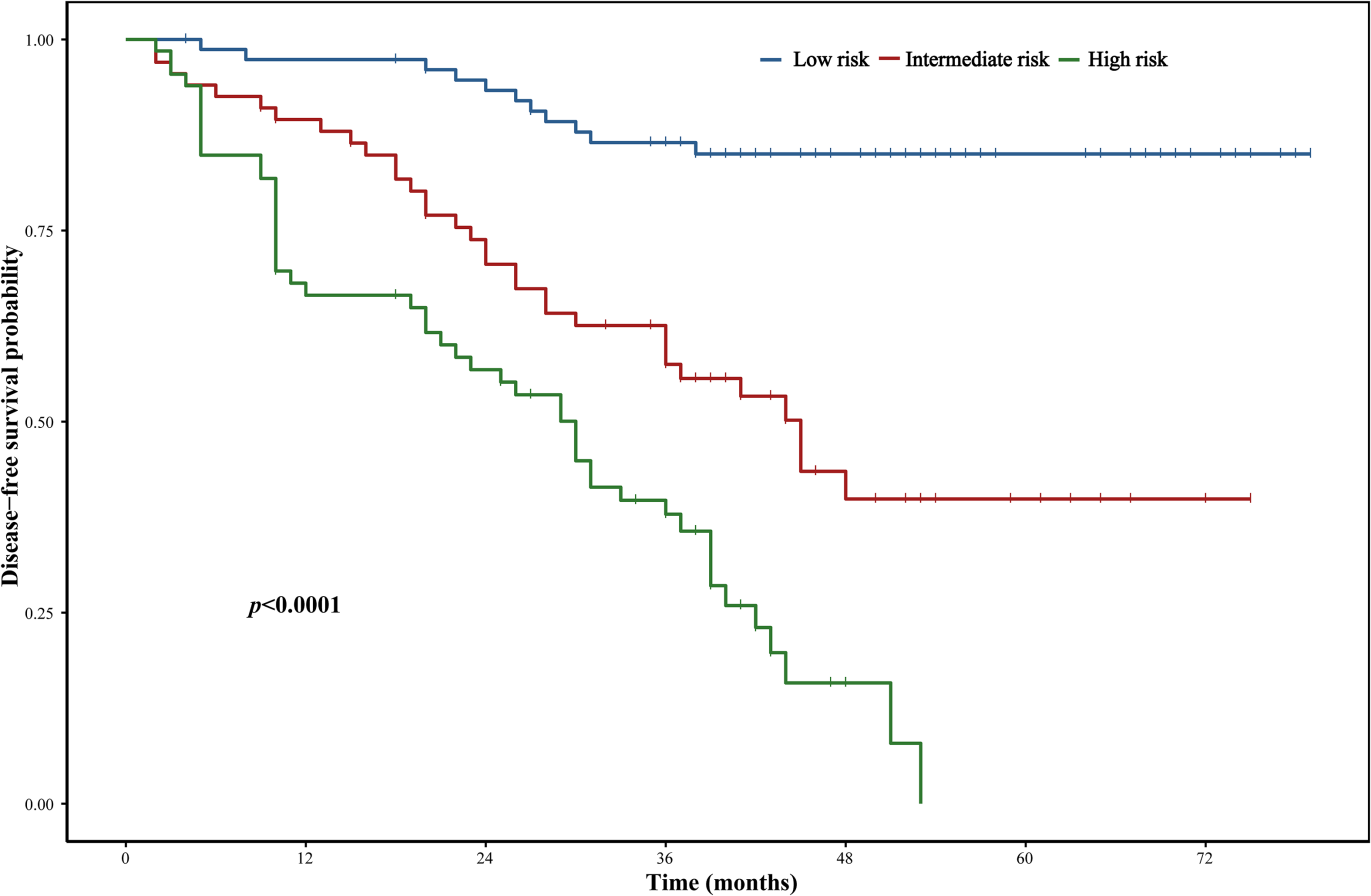
Risk stratification of patients with stage III colon cancer based on the prognostic model.

To enhance clinical applicability, patients were stratified into low-, intermediate-, and high-risk groups according to total nomogram score. Kaplan–Meier analysis showed significantly distinct DFS outcomes among the three groups, with clear separation of survival curves, as presented in **Figure 4**.

These findings indicate that integration of inflammatory indices with clinicopathological factors enables robust individualized recurrence risk prediction in stage III colon cancer.

## 4 Discussion

This study provides an integrated evaluation of preoperative inflammatory and immunonutritional indices in stage III colon cancer, spanning diagnostic discrimination, clinicopathological correlations, independent prognostic value, and individualized risk modeling. Compared with patients with benign colonic lesions, those with stage III colon cancer demonstrated significantly lower HALP and LMR values and higher SII levels, reflecting a systemic state characterized by inflammation, nutritional compromise, and immune imbalance. Such alterations are biologically plausible: tumor-associated cytokine activity can suppress hemoglobin and hepatic albumin synthesis, promote neutrophilia and thrombocytosis, and impair lymphocyte-mediated immune surveillance (9, 20, 21). Composite indices such as HALP and LMR therefore encapsulate multiple dimensions of host–tumor interaction rather than representing isolated laboratory abnormalities (22).

In exploratory analyzes, we observed that HALP, SII, and LMR exhibited moderate discriminatory ability in distinguishing malignant from benign colonic lesions. However, these findings should be interpreted with caution. Given their limited specificity and overlap with non-malignant inflammatory conditions, these biomarkers are not intended to serve as standalone diagnostic tools or to replace established diagnostic modalities such as endoscopy or histopathological evaluation. Instead, they may be more appropriately regarded as indicators of tumor-related systemic alterations, reflecting the underlying inflammatory and immunonutritional state of the host.

The prognostic analyzes further reinforced the clinical relevance of inflammatory indices. Low HALP, high SII, and low LMR were associated with inferior disease-free survival, with early separation of Kaplan–Meier curves after surgery, indicating that host systemic condition at diagnosis may influence early recurrence dynamics. Among these markers, LMR demonstrated the most consistent association, retaining significance for overall survival as well. In multivariable analysis, HALP and LMR remained independent predictors of disease-free survival together with tumor stage and chemotherapy cycles, whereas SII did not retain independent significance. This pattern suggests partial overlap between SII and other inflammatory components, while HALP and LMR may capture complementary aspects of systemic condition—namely nutritional–hematologic reserve and immune–myeloid balance (23–26). Although completion of adjuvant chemotherapy is known to be associated with improved outcomes, the number of cycles may also be influenced by treatment tolerance, toxicity, and early disease progression. Therefore, this variable likely reflects not only treatment exposure but also aspects of patient condition and disease course. In this context, the present model is best understood as a postoperative prognostic tool rather than a strictly baseline prediction model (27).

Building upon these findings, we developed a nomogram incorporating HALP, LMR, tumor stage, and chemotherapy cycles to predict individualized 1-, 2-, and 3-year DFS. The model demonstrated good discrimination and maintained stable accuracy in time-dependent ROC analyzes. Calibration curves showed close agreement between predicted and observed outcomes, and DCA suggested meaningful net clinical benefit compared with stage-based prediction alone. Importantly, stratification by total nomogram score separated patients into distinct low-, intermediate-, and high-risk groups with significantly different disease-free survival curves, underscoring the model’s potential practical value.

In terms of discrimination, the predictive performance observed in our study is comparable to previously reported clinicopathologic nomograms for resected non-metastatic colorectal cancer (28). For example, Dai et al. reported C-indices in the range of approximately 0.70–0.75 for stage I–III CRC prognostic models based on clinical variables (29). Similarly, radiomics-based nomograms for stage II–III colon cancer have achieved C-indices approaching 0.78 (30). While such advanced models may provide slightly higher discrimination, they typically require dedicated imaging feature extraction and specialized analytical platforms. In contrast, our model achieves competitive predictive accuracy using only routinely available laboratory and clinicopathological variables, thereby enhancing feasibility and applicability in everyday clinical practice.

Beyond its statistical performance, this nomogram may support more individualized postoperative management in stage III colon cancer. By integrating inflammatory markers with clinicopathological variables, it provides a practical estimate of recurrence risk that complements conventional stage-based assessment. Patients classified as high risk may require closer follow-up, particularly during the first two postoperative years when recurrence is most likely. In clinical practice, this may involve more frequent visits, imaging examinations, and closer monitoring of symptoms or tumor markers, along with greater attention to treatment adherence and overall condition. In contrast, patients at low risk may be managed with standard surveillance, potentially avoiding unnecessary investigations. For intermediate-risk patients, the model may offer additional support for tailoring follow-up intensity according to individual risk. As all variables are routinely available, the model can be readily applied in daily practice without additional cost. It is therefore best viewed as a complementary tool to assist risk-adapted surveillance rather than to replace existing follow-up strategies.

Several limitations should be acknowledged. This study was a retrospective single-center study, which may introduce selection bias and limit the generalizability of the findings. Although internal validation demonstrated stable model performance, external validation in independent and preferably multicenter cohorts is required to confirm its broader applicability. Therefore, the present model should be interpreted as a preliminary prognostic tool rather than a definitive clinical decision-making instrument. Molecular prognostic markers, such as microsatellite instability (MSI) status and other genomic alterations, were not incorporated into the current model. These factors have been shown to provide important prognostic information in colorectal cancer and may further enhance predictive performance. Future studies integrating molecular and clinicopathological variables may enable more comprehensive and precise risk stratification. Furthermore, the retrospective design precluded assessment of dynamic changes in inflammatory indices over time, which may also carry prognostic relevance and warrant further investigation.

In summary, this study demonstrates that preoperative HALP and LMR provide independent and clinically meaningful prognostic information beyond conventional staging in stage III colon cancer. By integrating these readily accessible inflammatory indices with tumor stage and chemotherapy exposure, we developed a practical nomogram capable of individualized recurrence risk estimation with stable predictive performance. Given that all included variables are derived from routine clinical assessments, this model may be readily implemented without additional cost or specialized testing. Such an approach supports a shift toward more refined, risk-adapted postoperative surveillance and management strategies. Further prospective and multicenter validation will help clarify its role within contemporary personalized treatment frameworks.

## 5 Conclusion

Compared with conventional TNM staging alone, the integration of HALP and LMR with TNM staging provides additional prognostic value. By reflecting the interplay between systemic inflammation, nutritional status, and immune surveillance, these readily available indices capture important aspects of host–tumor interaction that influence recurrence risk. The integrative nomogram incorporating HALP, LMR, tumor stage, and chemotherapy exposure enables individualized prediction of disease-free survival using routine clinical data. With further external validation, this accessible risk-stratification approach may facilitate more tailored postoperative surveillance and support treatment decision-making in real-world practice.

## Data Availability

The raw data supporting the conclusions of this article will be made available by the authors, without undue reservation.
