# Supplementary material for: Preoperative HALP and LMR predict disease-free survival in stage III colon cancer and enable nomogram-based risk stratification
Source: Front Oncol. 2026 Apr 29;16:1825101. doi: 10.3389/fonc.2026.1825101 (PMC13167495; doi:10.3389/fonc.2026.1825101)
Supplement: Supplementary file 1 [file DataSheet1.docx]

Supplementary Material

# Supplementary Figures and Tables

## Supplementary Figures

**Supplementary Figure 1.** Kaplan–Meier survival curves for disease-free survival and overall survival according to HALP, SII, and LMR in patients with stage III colon cancer.


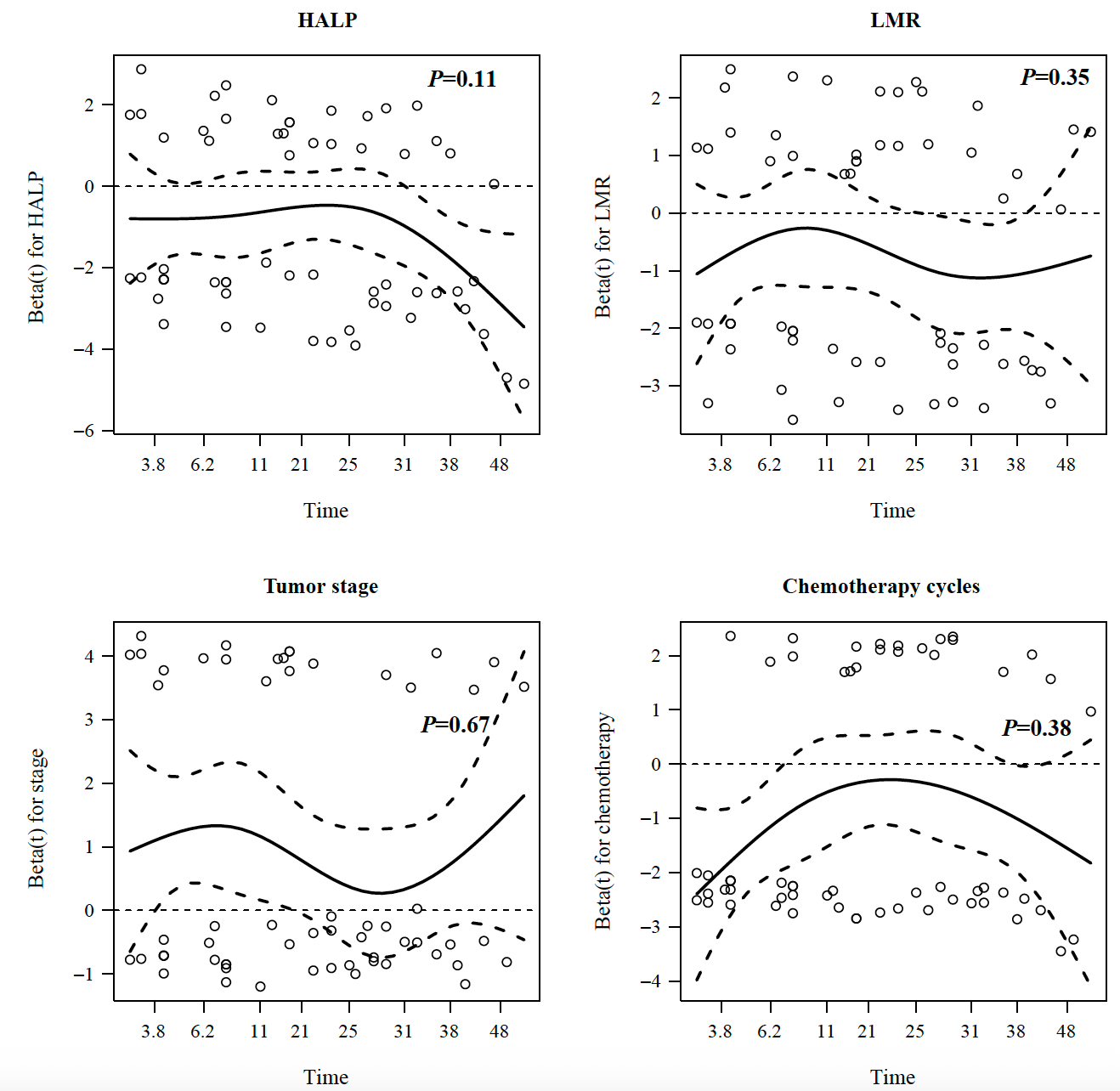


**Supplementary Figure 2.** Assessment of the proportional hazards assumption using Schoenfeld residuals for variables included in the Cox model.

## Supplementary Tables

Table S1 Clinic pathological Characteristics According to HALP Stratification

| Variables | Low HALP | High HALP | χ^2^ | *P* |
| --- | --- | --- | --- | --- |
| Age |  |  | 0.110 | 0.740 |
| ≤65 | 32（45.1%） | 66（47.5%） |  |  |
| >65 | 39（54.9%） | 73（52.5%） |  |  |
| Sex |  |  | 1.553 | 0.213 |
| Male | 36（50.7%） | 83（59.7%） |  |  |
| Female | 35（49.3%） | 56（40.3%） |  |  |
| Smoking History | 13（18.3%） | 37（26.6%） | 1.788 | 0.181 |
| Alcohol Consumption | 11（15.5%） | 31（22.3%） | 1.362 | 0.243 |
| Hypertension History | 16（22.5%） | 40（28.8%） | 0.936 | 0.333 |
| Diabetes History | 14（19.7%） | 29（20.9%） | 0.038 | 0.846 |
| BMI |  |  | 0.366 | 0.833 |
| <18.5 | 5（7.0%） | 8（5.8%） |  |  |
| 18.5-24.9 | 40（56.3%） | 84（60.4%） |  |  |
| ≥25.0 | 26（36.7%） | 47（33.8%） |  |  |
| Surgical Approach |  |  | 0.050 | 0.824 |
| Laparoscopic | 49（69.0%） | 98（70.5%） |  |  |
| Open Surgery | 22（31.0%） | 41（29.5%） |  |  |
| Preoperative Obstruction | 22（31.0%） | 24（17.3%） | 5.171 | 0.023 |
| Tumor Differentiation |  |  | 4.757 | 0.029 |
| Well/Moderately | 52（73.2%） | 119（85.6%） |  |  |
| Poor | 19（26.8%） | 20（14.4%） |  |  |
| Maximum Tumor Diameter |  |  | 12.125 | <0.001 |
| <5cm | 27（38.0%） | 88（63.3%） |  |  |
| ≥5cm | 44（62.0%） | 51（36.7%） |  |  |
| Depth of Invasion |  |  | 8.214 | 0.016 |
| T1-2 | 0（0.0%） | 9（6.5%） |  |  |
| T3 | 40（56.3%） | 90（64.7%） |  |  |
| T4 | 31（43.7%） | 40（28.8%） |  |  |
| Lymph Node Metastasis |  |  | 2.739 | 0.098 |
| N1 | 53（74.6%） | 88（63.3%） |  |  |
| N2 | 18（25.4%） | 51（36.7%） |  |  |
| Tumor Stage |  |  | 0.038 | 0.846 |
| IIIA-IIIB | 57（80.3%） | 110（79.1%） |  |  |
| IIIC | 14（19.7%） | 29（20.9%） |  |  |
| Vascular Invasion | 35（49.3%） | 74（53.2%） | 0.292 | 0.589 |
| Perineural Invasion | 31（43.7%） | 41（29.5%） | 4.186 | 0.041 |
| Tumor Location |  |  | 0.647 | 0.421 |
| Left-sided | 43（60.6%） | 92（66.2%） |  |  |
| Right-sided | 28（39.4%） | 47（33.8%） |  |  |
| Number of Chemotherapy Cycles |  |  | 0.647 | 0.421 |
| <6 | 43（60.6%） | 92（66.2%） |  |  |
| 6-8 | 28（39.4%） | 47（33.8%） |  |  |
| CEA |  |  | 7.314 | 0.007 |
| <5 | 30（42.3%） | 86（61.9%） |  |  |
| ≥5 | 41（57.7%） | 53（38.1%） |  |  |
| CA199 |  |  | 3.841 | 0.063 |
| <37 | 41（57.7%） | 99（71.2%） |  |  |
| ≥37 | 30（42.3%） | 40（28.8%） |  |  |

Table S2 Clinic pathological Characteristics According to SII Stratification

| Variables | Low SII | High SII | χ^2^ | *P* |
| --- | --- | --- | --- | --- |
| Age |  |  | <0.001 | 1.000 |
| ≤65 | 56（46.7%） | 42（46.7%） |  |  |
| >65 | 64（53.3%） | 48（53.3%） |  |  |
| Sex |  |  | 0.079 | 0.778 |
| Male | 67（55.8%） | 52（57.8%） |  |  |
| Female | 53（44.2%） | 38（42.2%） |  |  |
| Smoking History | 26（21.7%） | 24（26.7%） | 0.709 | 0.400 |
| Alcohol Consumption | 24（20.0%） | 18（20.0%） | 0.000 | 1.000 |
| Hypertension History | 28（23.3%） | 28（31.1%） | 1.591 | 0.207 |
| Diabetes History | 21（17.5%） | 22（24.4%） | 1.523 | 0.217 |
| BMI |  |  | 1.131 | 0.568 |
| <18.5 | 6（5.0%） | 7（7.8%） |  |  |
| 18.5-24.9 | 74（61.7%） | 50（55.6%） |  |  |
| ≥25.0 | 40（33.3%） | 33（36.7%） |  |  |
| Surgical Approach |  |  | 2.315 | 0.128 |
| Laparoscopic | 89（74.2%） | 58（64.4%） |  |  |
| Open Surgery | 31（25.8%） | 32（35.6%） |  |  |
| Preoperative Obstruction | 19（15.8%） | 27（30.0%） | 6.034 | 0.014 |
| Tumor Differentiation |  |  | 3.592 | 0.058 |
| Well/Moderately | 103（85.8%） | 68（75.6%） |  |  |
| Poor | 17（14.2%） | 22（24.4%） |  |  |
| Maximum Tumor Diameter |  |  | 4.166 | 0.041 |
| <5cm | 73（60.8%） | 42（46.7%） |  |  |
| ≥5cm | 47（39.2%） | 48（53.3%） |  |  |
| Depth of Invasion |  |  | 1.636 | 0.441 |
| T1-2 | 7（5.8%） | 2（2.2%） |  |  |
| T3 | 73（60.8%） | 57（63.3%） |  |  |
| T4 | 40（33.3%） | 31（34.4%） |  |  |
| Lymph Node Metastasis |  |  | 0.016 | 0.899 |
| N1 | 81（67.5%） | 60（66.7%） |  |  |
| N2 | 39（32.5%） | 30（33.3%） |  |  |
| Tumor Stage |  |  | 0.039 | 0.843 |
| IIIA-IIIB | 96（80.0%） | 71（78.9%） |  |  |
| IIIC | 24（20.0%） | 19（21.1%） |  |  |
| Vascular Invasion | 64（53.3%） | 45（50.0%） | 0.229 | 0.632 |
| Perineural Invasion | 38（31.7%） | 34（37.8%） | 0.852 | 0.356 |
| Tumor Location |  |  | 0.002 | 0.967 |
| Left-sided | 77（64.2%） | 58（64.4%） |  |  |
| Right-sided | 43（35.8%） | 32（35.6%） |  |  |
| Number of Chemotherapy Cycles |  |  | 2.422 | 0.120 |
| <6 | 51（42.5%） | 48（53.3%） |  |  |
| 6-8 | 69（57.5%） | 42（46.7%） |  |  |
| CEA |  |  | 2.568 | 0.109 |
| <5 | 72（60.0%） | 44（48.9%） |  |  |
| ≥5 | 48（40.0%） | 46（51.1%） |  |  |
| CA199 |  |  | 0.350 | 0.554 |
| <37 | 82（68.3%） | 58（64.4%） |  |  |
| ≥37 | 38（31.7%） | 32（35.6%） |  |  |

Table S3 Clinic pathological Characteristics According to LMR Stratification

| Variables | Low LMR | High LMR | χ^2^ | *P* |
| --- | --- | --- | --- | --- |
| Age |  |  | 0.116 | 0.733 |
| ≤65 | 39（48.1%） | 59（45.7%） |  |  |
| >65 | 42（51.9%） | 70（54.3%） |  |  |
| Sex |  |  | 2.129 | 0.145 |
| Male | 51（63.0%） | 68（52.7%） |  |  |
| Female | 30（37.0%） | 61（47.3%） |  |  |
| Smoking History | 22（27.2%） | 28（21.7%） | 0.816 | 0.366 |
| Alcohol Consumption | 20（24.7%） | 22（17.1%） | 1.814 | 0.178 |
| Hypertension History | 22（27.2%） | 34（26.4%） | 0.016 | 0.898 |
| Diabetes History | 18（22.2%） | 25（19.4%） | 0.247 | 0.619 |
| BMI |  |  | 0.065 | 0.968 |
| <18.5 | 5（6.2%） | 8（6.2%） |  |  |
| 18.5-24.9 | 47（58.0%） | 77（59.7%） |  |  |
| ≥25.0 | 29（35.8%） | 44（34.1%） |  |  |
| Surgical Approach |  |  | 13.101 | <0.001 |
| Laparoscopic | 45（55.6%） | 102（79.1%） |  |  |
| Open Surgery | 36（44.4%） | 27（20.9%） |  |  |
| Preoperative Obstruction | 24（29.6%） | 22（17.1%） | 4.600 | 0.032 |
| Tumor Differentiation |  |  | 6.433 | 0.011 |
| Well/Moderately | 59（72.8%） | 112（86.8%） |  |  |
| Poor | 22（27.2%） | 17（13.2%） |  |  |
| Maximum Tumor Diameter |  |  | 2.328 | 0.127 |
| <5cm | 39（48.1%） | 76（58.9%） |  |  |
| ≥5cm | 42（51.9%） | 53（41.1%） |  |  |
| Depth of Invasion |  |  | 3.461 | 0.177 |
| T1-2 | 1（1.2%） | 8（6.2%） |  |  |
| T3 | 54（66.7%） | 76（58.9%） |  |  |
| T4 | 26（32.1%） | 45（34.9%） |  |  |
| Lymph Node Metastasis |  |  | 0.237 | 0.626 |
| N1 | 56（69.1%） | 85（65.9%） |  |  |
| N2 | 25（30.9%） | 44（34.1%） |  |  |
| Tumor Stage |  |  | 0.825 | 0.364 |
| IIIA-IIIB | 67（82.7%） | 100（77.5%） |  |  |
| IIIC | 14（17.3%） | 29（22.5%） |  |  |
| Vascular Invasion | 42（51.9%） | 67（51.9%） | <0.001 | 0.990 |
| Perineural Invasion | 41（50.6%） | 31（24.0%） | 15.610 | <0.001 |
| Tumor Location |  |  | 0.376 | 0.540 |
| Left-sided | 50（61.7%） | 85（65.9%） |  |  |
| Right-sided | 31（38.3%） | 44（34.1%） |  |  |
| Number of Chemotherapy Cycles |  |  | 0.639 | 0.424 |
| <6 | 41（50.6%） | 58（45.0%） |  |  |
| 6-8 | 40（49.4%） | 71（55.0%） |  |  |
| CEA |  |  | 0.247 | 0.619 |
| <5 | 43（53.1%） | 73（56.6%） |  |  |
| ≥5 | 38（46.9%） | 56（43.4%） |  |  |
| CA199 |  |  | 0.814 | 0.367 |
| <37 | 57（70.4%） | 83（64.3%） |  |  |
| ≥37 | 24（29.6%） | 46（35.7%） |  |  |

Table S4. Cox Regression Analysis of Prognostic Factors for DFS (In training set)

| Covariates | Univariate analysis | | | Multivariate analysis | | | |  |
| --- | --- | --- | --- | --- | --- | --- | --- | --- |
|  | HR | 95%CI | *P* | | HR | 95%CI | *P* | |
| Sex |  |  |  | |  |  |  | |
| Male | Reference |  |  | |  |  |  | |
| Female | 0.636 | 0.370-1.092 | 0.101 | |  |  |  | |
| Age |  |  |  | |  |  |  | |
| ≤65 | Reference |  |  | |  |  |  | |
| >65 | 0.943 | 0.573-1.552 | 0.818 | |  |  |  | |
| Smoking History | 1.008 | 0.571-1.781 | 0.978 | |  |  |  | |
| Alcohol Consumption | 1.004 | 0.553-1.821 | 0.990 | |  |  |  | |
| Hypertension History | 1.033 | 0.591-1.805 | 0.910 | |  |  |  | |
| Diabetes History | 1.188 | 0.672-2.100 | 0.553 | |  |  |  | |
| BMI |  |  | 0.812 | |  |  |  | |
| <18.5 | Reference |  |  | |  |  |  | |
| 18.5-24.9 | 0.743 | 0.264-2.091 | 0.574 | |  |  |  | |
| ≥25.0 | 0.701 | 0.238-2.062 | 0.518 | |  |  |  | |
| Surgical Approach |  |  |  | |  |  |  | |
| Laparoscopic | Reference |  |  | |  |  |  | |
| Open Surgery | 1.167 | 0.680-2.003 | 0.575 | |  |  |  | |
| Preoperative Obstruction | 2.509 | 1.439-4.373 | 0.001 | |  |  |  | |
| Tumor Differentiation |  |  |  | |  |  |  | |
| Well / Moderately | Reference |  |  | |  |  |  | |
| Poor | 2.620 | 1.523-4.507 | <0.001 | |  |  |  | |
| Maximum Tumor Diameter |  |  |  | |  |  |  | |
| <5cm | Reference |  |  | |  |  |  | |
| ≥5cm | 1.743 | 1.048-2.898 | 0.032 | |  |  |  | |
| Tumor Stage |  |  |  | |  |  |  | |
| IIIA-IIIB | Reference |  |  | | Reference |  |  | |
| IIIC | 2.474 | 1.460-4.191 | <0.001 | | 2.435 | 1.432-4.140 | 0.001 | |
| Vascular Invasion | 0.932 | 0.566-1.534 | 0.781 | |  |  |  | |
| Perineural Invasion | 2.122 | 1.286-3.502 | 0.003 | |  |  |  | |
| Tumor Location |  |  |  | |  |  |  | |
| Left-sided | Reference |  |  | |  |  |  | |
| Right-sided | 1.435 | 0.870-2.368 | 0.158 | |  |  |  | |
| Number of Chemotherapy Cycles |  |  |  | |  |  |  | |
| <6 | Reference |  |  | | Reference |  |  | |
| ≥6 | 0.347 | 0.204-0.590 | <0.001 | | 0.380 | 0.223-0.647 | <0.001 | |
| HALP |  |  |  | |  |  |  | |
| ≤24.11 | Reference |  |  | | Reference |  |  | |
| >24.11 | 0.286 | 0.172-0.476 | <0.001 | | 0.384 | 0.225-0.655 | <0.001 | |
| SII |  |  |  | |  |  |  | |
| ≤844.81 | Reference |  |  | |  |  |  | |
| >844.81 | 2.007 | 1.216-3.310 | 0.006 | |  |  |  | |
| LMR |  |  |  | |  |  |  | |
| ≤2.32 | Reference |  |  | | Reference |  |  | |
| >2.32 | 0.371 | 0.224-0.614 | <0.001 | | 0.483 | 0.286-0.815 | 0.006 | |
| CEA |  |  |  | |  |  |  | |
| <5 | Reference |  |  | |  |  |  | |
| ≥5 | 1.484 | 0.900-2.447 | 0.122 | |  |  |  | |
| CA199 |  |  |  | |  |  |  | |
| <37 | Reference |  |  | |  |  |  | |
| ≥37 | 1.479 | 0.897-2.438 | 0.125 | |  |  |  | |
